# An Investigation of the Prescription Patterns of Chinese Herbal Products for Chronic Glomerulonephritis Patients: A Hospital-Based Cross-Sectional Study

**DOI:** 10.1155/2018/5080764

**Published:** 2018-11-15

**Authors:** Wen Chen, Hsing-Yu Chen, Yao-Hsu Yang, Sien-Hung Yang, Ching-Wei Yang, You-Hung Wu, Jiun-Liang Chen

**Affiliations:** ^1^Division of Chinese Internal Medicine, Center for Traditional Chinese Medicine, Chang Gung Memorial Hospital, Taoyuan, Taiwan; ^2^School of Chinese Medicine, China Medical University, Taichung, Taiwan; ^3^School of Traditional Chinese Medicine, College of Medicine, Chang Gung University, Taoyuan, Taiwan; ^4^Graduate Institute of Clinical Medical Sciences, College of Medicine, Chang Gung University, Taoyuan, Taiwan; ^5^Department for Traditional Chinese Medicine, Chang Gung Memorial Hospital, Chia-Yi, Taiwan; ^6^Institute of Occupational Medicine and Industrial Hygiene, National Taiwan University College of Public Health, Taipei, Taiwan; ^7^Center of Excellence for Chang Gung Research Data link, Chang Gung Memorial Hospital, Chia-Yi, Taiwan; ^8^Chang Gung Immunology Consortium, Chang Gung Memorial Hospital and Chang Gung University, Gueishan, Taoyuan, Taiwan; ^9^Graduate Institute of Acupuncture Science, College of Medicine, China Medical University, Taichung, Taiwan

## Abstract

Chronic kidney disease (CKD) has a high incidence and prevalence worldwide, and chronic glomerulonephritis (CGN) is one of the main causes of CKD. Therefore, it is important to diagnose and treat CGN early. The purpose of this study is to analyze the prescription patterns and frequencies of Chinese herbal products (CHPs) for CGN by using a hospital-based database from the Chang Gung Memorial Hospital (CGMH), a large, tertiary hospital system in Taiwan, and to evaluate the safety and possible efficacy of CHPs by blood test. The International Classification of Disease Ninth Revision (ICD-9) code 582 was used to identify patients with CGN. From 2004 to 2015, a total of 54726 CHP prescriptions for CGN were provided. Association rule mining was used to analyze the prevalent of CHP combination patterns in treating CGN. Jia-Wei-Xiao-Yao-San (JWXYS) and* Gorgon (Euryale feroxSalisb.)* were the most frequently prescribed herbal formula (HF) and single herb (SH), respectively. The most frequently prescribed combination of CHPs was that of JWXYS with Bu-Yang-Huan-Wu-Tang (BYHWT) in CGMH. In statistical, the level of eGFR in Stage 3a and 3b group was increasing after treatment in 6 and 12 months and might not cause the renal function to worsen within 12-month treatments. To the best of our knowledge, this is the first pharmacoepidemiological study to review CHP treatments for CGN. However, additional studies and clinical trials are needed to provide data on the safety and efficacy of these CHPs.

## 1. Introduction

In the aging society, the proportion of patients with hypertension, diabetes, and other chronic diseases is increasing. The incidence and prevalence of chronic kidney disease (CKD) are very high worldwide [1]. There are no obvious symptoms of CKD, so patients are not aware that they have CKD. According to a study from the Taiwan National Institutes of Health in 2008, 11.9% of people over the age of 20 in Taiwan had CKD, but only approximately 10% of them knew that they were suffering from kidney disease [2]. CKD was defined by the National Kidney Foundation Kidney Disease Outcomes Quality Initiative, and the definition was modified by the international guidelines of a group known as the Kidney Disease Improving Global Outcomes. The diagnostic standards of CKD include kidney dysfunction or structural damage over a period of three months, and an impact on health. In the Kidney Disease Outcomes Quality Initiative research in 2012, the most common causes of CKD were chronic glomerulonephritis (CGN), diabetic nephropathy, and hypertensive renal sclerosis [3]. Caring for patients with CKD is a big burden for the national economy. Therefore, early diagnosis, treatment, and prevention as well as slowing the progression of CKD and CGN are very important.

CGN is a group of diseases that occur in the glomerulus. The mechanism of CGN is considered to be a series of inflammatory reactions caused by complex deposition that activate the complement system. There are many causes of CGN, such as bacterial and viral infections, immune responses by other systemic diseases, environmental toxins, drugs, cancers, thyroid disease, diabetes, and hepatitis B [4].

The current medications for CKD in patients with drug treatment are focused on drugs that slow down the residual renal function and those that avoid residual renal function deterioration and side effects. Treatment for CGN can include steroids and immunosuppressive agents, and partial patients can use angiotensin converting enzyme inhibitors (ACEi) and angiotensin II receptor blockers (ARB) to relieve microvascular pressure in the kidney to reduce proteinuria and improve CKD. However, the clinical applications of CGN drugs are limited by numerous adverse side effects such as poor control of diabetes mellitus, high levels of lipids in the blood, hypertension, insomnia, obesity, edema, osteoporosis, peptic ulcer, infection, hyperkalemia, and angioedema [4, 5].

In recent years, complementary and alternative therapies have become increasingly popular for their potential efficacy and a small number of side effects for treating CKD [6–8]. The herbal formula (HF) known as Liu-Wei-Di-Huang-Wan (LWDHW) is reported to reduce the risk of dialysis and prolong the time to dialysis in CKD patients [6]. The CHPs of wind dampness-dispelling formulas and harmonizing formulas may have the effect of protecting kidney function before dialysis [7]. In CGMH, research has found that combinations of Jia-Wei-Xiao-Yao-San (JWXYS) and Bu-Yang-Huan-Wu-Tang (BYHWT) may improve the quality of life in CKD patients [8]. According to the traditional Chinese medicine (TCM) theory, TCM physicians evaluate the conditions of patients to prescribe one or more herbal formulas combined with several SHs for each prescription. However, few studies have reported on CGN in TCM. The aim of this study is to analyze the CHPs prescribed and the coprescription patterns for treating CGNs by using a hospital-based database and follow up the eGFR to evaluate the safety and possibly efficacy of CHPs coprescription patterns. Our hope is that these findings can provide reliable information on adjunctive therapy for CGN in the future.

## 2. Material and Methods

### 2.1. Data Source

We analyzed a sample of patients from CGMH Taipei, Linkou, Taoyuan Districts in Taiwan and determined the prevalence of prescribed CHPs in patients with CGN from 2004 to 2015. The electronic database of all claims obtained from the CGMH website included medical record files containing the patient's gender and date of birth, date of medical visits, medical care facilities and specialties, prescription drugs, management and treatment, unique identification numbers, and all diagnoses coded according to the International Classification of Diseases, Ninth Revision, Clinical Modification (ICD-9-CM) format.

### 2.2. Study Subjects

The study cohort comprised patients diagnosed with CGN (ICD-9-CM codes:582) from 2004 to 2015; information was obtained from TCM outpatient visit records. All CGN-related medical records were analyzed during the study period. In the CGN cohort, patients who had at least one CHP were defined as CHPs users. Those who underwent kidney dialysis treatment were excluded. Patients accepted blood test before treatment and after treatments for 6 and 12 months, totally three times. We observe eGFR in blood according to different stages: Stage 1-2 as eGFR ≥ 60 mL/min/1.73m^2^, Stage 3a as eGFR 45-59 mL/min/1.73m^2^, Stage 3b as eGFR44-30 mL/min/1.73m^2^, Stage 4 as eGFR 29-15 mL/min/1.73m^2^, and Stage 5 as eGFR< 15 mL/min/1.73m^2^.

### 2.3. Study Variables

Information on patient characteristics included the patient's age, gender, and comorbidities. Patients were divided into three age groups as follows:<35years old, 35-55 years old, and >55 years old. Comorbidities commonly seen in kidney disease patients were chosen, including hypertension, diabetes, hyperlipidemia, gout, cardiovascular disease, ischemic heart disease, atherosclerosis, diseases of the digestive system, systemic lupus erythematosus, and rheumatoid arthritis. These comorbidities were considered as covariates in modeling.

Prevalence visits and average daily dose (g) of CHPs contained in prescriptions were the variables used in this study to identify single agents and combinations of CHPs commonly used for CGN. Quality control of CHPs is assured as SHs and HFs are all produced as concentrated powders by Good Manufacturing Practice pharmaceutical factories with advantages of high safety and stability, convenient use, and reduction in material change problems caused by poor storage.

### 2.4. Ethics Statement

We obtained ethical approval for this study from the Institutional Review Board of the Chang Gung Memorial Foundation (CGMF) (IRB No: 201601457B0). Since deidentified data were used, a waiver of informed consent was granted by the Institutional Review Board of the CGMF.

### 2.5. Statistical Analysis

Statistical analysis was performed using SAS Enterprise Guide 4.3 (SAS Institute Inc., Cary, NC, USA), which was used for data linkage and descriptive statistical analysis of drug utilization patterns. Figures were created using Sigma Plot 12.0 (Systat Software Inc., USA). Using paired-samples t test and P<0.05 indicated statistical significance between groups. TCM doctors commonly wrote a prescription of more than two CHPs to treat patients. Association rule mining (ARM) was used in this study to explore the combination patterns of CHPs frequently used for CGN.

## 3. Results

### 3.1. Demographic Characteristics of CHPs Users

We used the data from 2004 to 2015 of patients who had an ICD-9 code of 582 from the Taipei, Linkou, and Taoyuan Chang-gung districts. A total of 54726 CHP prescriptions used by 4438 CHPs users were included in this study. Patient demographic included age, gender, and potential comorbidities related to CGN, as shown in Table 1. The common comorbidities of patients with CGN receiving CHPs treatment were diseases of the digestive system, hypertension, diabetes mellitus, and hyperlipidemia.

### 3.2. The Most Commonly Used HF, SH, and Combinations of Two CHPs

**Figure 1:**
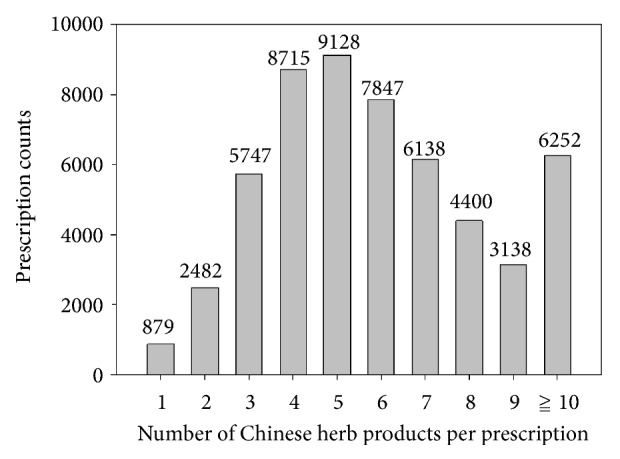
**Distribution of Chinese herbal products (CHPs) counts per prescription.** TCM doctors commonly prescribe 5 CHPs per prescription and, on average, each prescription contained 6.04 CHPs for chronic glomerulonephritis patients. A single CHP in one prescription is the least frequent.

A total of 470 different CHPs were used and, on average, each prescription contained 6.04 CHPs.  Figure 1 shows that TCM doctors commonly prescribe 5 CHPs, and more than 90 percent of prescriptions contained more than two CHPs.

Tables 2 and 3 present the most frequently prescribed herbal formulas and single herbs during these outpatient visits. JWXYS was the most commonly used HF (60.2%), followed by Bu-Yang-Huan-Wu-Tang (BYHWT) (37.3%) and Du-Huo-Ji-Sheng-Tang (19.0%)(Table 2). Among the single herb,* Euryale ferox Salisb. *was prescribed most frequently (10.0%), followed by* Salvia miltiorrhiza Bunge* (8.2%) and* Coix lacryma-jobi var.ma-yuen (Rom. Caill.) Stapf *(7.2%) (Table 3).

According to the association rules in Table 4, the most commonly prescribed combination of two CHPs for CGN was that of Jia-Wei-Xiao-Yao-San with Bu-Yang-Huan-Wu-Tang, followed by Jia-Wei-Xiao-Yao-San with Du-Huo-Ji- Sheng-Tang and Jia-Wei-Xiao-Yao-San with Ching-Shin- Lian-Tzyy- Yinn.

### 3.3. Core Treatment for CGN

**Figure 2:**
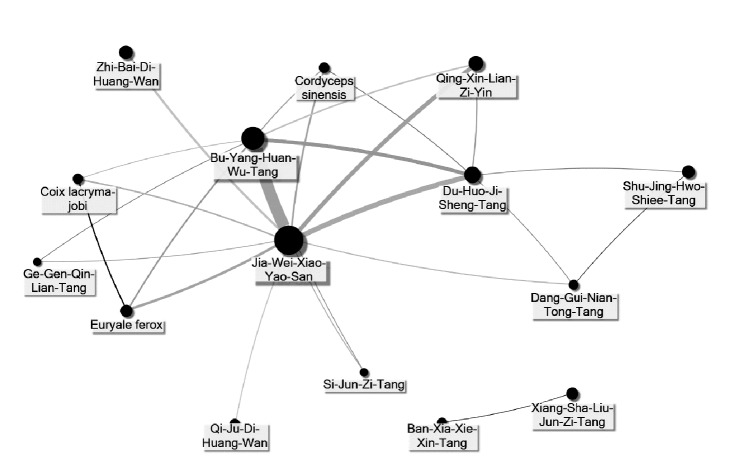
**Social network analysis on associations between commonly used CHPs for CGN**. JWXYS was the center of the CHPs network prescribed for CGN and became the core treatment for CGN. Larger circle represents higher frequency of prescription, and thicker connection line represents more common combination.

JWXYS was the core treatment for CGN because it accounted for the highest prevalence among all CHPs. Additionally, the central role of JWXYS can be found in the network of commonly used coprescriptions of CHPs (Figure 2).

### 3.4. Modern Blood Biochemical Test

**Figure 3:**
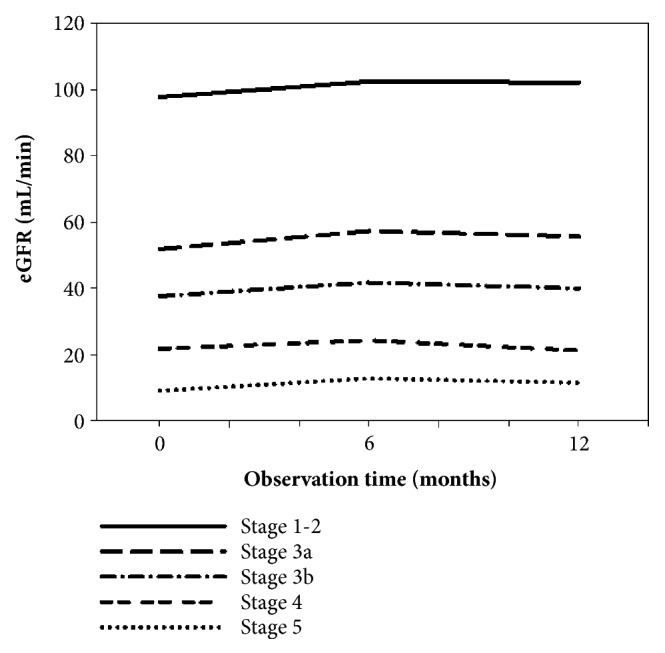
T**he level ofeGFR in CGN patients with CHPs.** Data was collected from Chang Gung Research Database. Chronic glomerulonephritis patients were selected as described under materials and methods (eGFR n =386). Figure 3 is the eGFR change according to the eGFR level before the initial treatment. All groups showed a significant upward trend within 6 months after CHPs use. Only the Stage 3a and 3b groups continued to increase significantly within 12 months.

According to the above, the most commonly prescribed combination of two herbal formulas for CGN was JWXYS with BYHWT. In this study, 386 patients have taken JWXYS and BYHWT and accepted blood tests at least three times. According to different eGFR stages, we observe blood tests before treatment and after treatments for 6 and 12 months. Data analysis and chart are in Table 5 and Figure 3.

## 4. Discussion

This study is the first hospital-based cross-sectional study on CGN with CHPs treatment and did not include utilization of health services provided by other hospitals or clinics. By using a hospital-based database, we can not only collect all of the prescriptions but also ensure the quality of CHPs. In addition, the detection of comorbidities is more extensive. Comorbidities with CGN, such as diabetes, hypertension, and cardiovascular diseases, may affect each other and induce many side effects during treatment. Therefore, doctors also relieve patients' comorbidities and uncomfortable symptoms, such as loss of appetite, nausea, vomiting, fatigue, and edema and improve the quality of daily life.

### 4.1. Commonly Used Herbal Formulas and Combinations of CHP for CGN

JWXYS in modern pharmacological research has been found to have anti-inflammatory, antitumor, antifibrosis, anticoagulant, and antiatherosclerotic effects. JWXYS has also been found to regulate immunity, reduce glycemic and lipid levels, induce analgesia, relieve emotional and neuropsychological disorders, and inhibit the central nervous system [29–34]. The antifibrotic effects and reduction in glycemic and lipid levels may improve kidney function by protecting blood vessels and microcirculation. CKD Stage 5 patients have high tendency of depression [35, 36]. JWXYS has been shown to have antidepressive and antianxiety effects, which may help patients stabilize their emotions and improve their quality of life [37]. Some CKD and CGN patients have digestive syndromes such as a loss of appetite, nausea, and vomiting. The incidence of gastrointestinal (GI) symptoms in patients with renal dialysis has been found to be high, and patients can have nonulcerative and nonvaricose GI bleeding [38]. JWXYS may also regulate GI function to relieve symptoms. In the Chinese medicine theory, JWXYS is used in patients with the pattern of liver depression and spleen deficiency and transforms into heat. Indications include emotional disorders, GI problems, insomnia, palpitations, upset, dry mouth, and difficulty and pain in micturition. Clear studies on the relationship between JWXYS and CKD and CGN are still lacking. However, the eGFR has an increasing trend in Stage 3a and 3b group in this study. Therefore, JWXYS may be chosen as adjuvant therapy for Stage 3a and 3b group in CKD and CGN based on the above research and can be used in the future to do more complete and rigorous clinical trials to evaluate the effects on renal function and find the possible mechanisms.

Modern pharmacological research has found that BYHWT may protect nerves and vascular endothelial cells, regulate immunity, have anti-inflammatory, antiplatelet aggregation, and antithrombotic effects, expand peripheral blood vessels, improve hemodynamics, and promote microcirculation [39–43]. BYHWT may regulate the performance of ICAM-1and VCAM-1 in diabetic mice to relieve inflammation and may reduce the levels of nitric oxide (NO). Therefore, BYHWT could improve renal microcirculation and alleviate pathological renal damage [19, 20].The main ingredient of BYHWT, Astragalus membranaceus (Fisch.) Bge., may have effects on reducing blood lipid levels and platelet aggregation rates and can also inhibit thrombosis by regulating NO synthase expression and inhibiting endothelial NO synthesis in some studies. BYHWT may also improve renal hemodynamics, protect renal function, and enhance the immune response that could reduce urine protein [11, 12, 44]. This finding may also be the potential reason for high rates of BYHWT use in CKD and CGN. In the Chinese medicine theory, BYHWT is used in patients with the pattern of qi deficiency with blood stasis. Indications include fatigue, micturition, enuresis, hemiplegia, and aphasia after stroke. A few studies have indicated that BYHWT can improve renal circulation and reduce urine protein, but these findings need more evidence to confirm the efficacy and possible mechanisms in the future.

JWXYS used with BYHWT is an example of a pair of HFs that increase treatment efficacy, which may be due to the effect of the two drugs acting through different modern pharmacology mechanisms to improve renal microcirculation and protect the kidneys. This theory may explain why JWXYS is used in combination with BYHWT and why they are the two most frequently prescribed HFs in combination to treat CGN.

### 4.2. Commonly Used Single Herb for CGN

*Salvia miltiorrhiza Bunge*,* Astragalus membranaceus*, and* Rheum Palmatum L.* are some of the top 10 most commonly used SHs.* Angelica sinensis (Oliv.) Diels and Ligusticum striatum DC. *are some ingredients of top ten HFs. These five SHs have effects such as antioxidation, antilipid peroxidation, antiplatelet aggregation, anti-inflammatory and antifibrotic effects, immune regulation, and regulation of renal vessels [45]. The pharmacological mechanisms of these findings may prevent the deterioration of CKD.

### 4.3. Modern Blood Biochemical Analysis

In the study, the level of eGFR was significantly increasing within 6 and 12 months in statistics for Stage 3a and 3b group. However, only within 6 months it had significant increase in statistics for Stage 4 and 5 group. This finding for Stage 4 and 5 patients might have association with their renal function decline, disease progression, or using more drugs leading to drug interaction.

In this study, we observe that patients in Stage 3a and 3b with CHPs might not decrease eGFR level group. It is presumed that the use of CHPs treatment within 12 months may be safe and might not cause the renal function to worsen in Stage 3a and 3b group. However, more researches are needed in the future.

### 4.4. Effectiveness and Safety of CHPs

Effectiveness and safety are the two important goals of drug use. The safety evaluation of CHPs often comes from personal observations, attempts, and clinical experience by ancient Chinese medical scientists and now could also be evaluated by modern pharmacological research and blood biochemical test.  Table 6 shows the analysis of commonly used CGN treatments in CHPs pharmacological research, and there were no uncomfortable symptoms found. However, further research is needed in the future to assess the safety and effectiveness of CHPs.

### 4.5. Limitations

There are some limitations to our study. First, this study restrictively used ICD-9 code 582 as the means of enrolling subjects only in the Chinese outpatient setting. We do not compare with the western medicine group. Meanwhile, we do not discuss whether the use of western medicine and the CHPs at the same time may affect the changes in the blood biochemical analysis, causing deviations in the evaluation of the results. Therefore, further well-designed, randomized, double-blinded, and placebo-controlled clinical trials are still needed to elucidate this problem.

Second, this study could not track the health outcomes of patients over time and could not rule out whether patients had other comorbidities along with CGN, including chronic diseases, pain, colds, or other complications. Whether these findings would influence the renal function and the data analysis on CHPs is unclear. Therefore, further studies that evaluate the efficacy and safety of CHPs in the CGN population are needed.

## 5. Conclusions

In conclusion, the most commonly prescribed combination for CGN in CGMH was that of JWXYS with BYHWT. After CHPs treatments, Stage 3a and 3b patients' eGFR level was increasing within 6 and 12 months and might not cause the renal function to worsen within 12 months treatments, but these still needed more evidences to confirm. The results of this study provide several directions for further cohort studies to elucidate the efficacy, safety, and drug-herb interactions of these commonly used CHPs among CGN patients.

## Figures and Tables

**Table 1:** Characteristics of chronic glomerulonephritis CHPs users from 2004 to 2015 at CGMH.(n=4438).

**Characteristics**	**CHPs users**	
	No.	%
**Age**		
<35	548	12.3
35-55	1383	31.2
>55	2507	56.5
**Gender**		
Female	1994	44.9
Male	2444	55.1
**Comorbidity with chronic glomerulonephritis (ICD-9-CM)**		
Hypertension (401-405)	374	8.4
Type II Diabetes Mellitus (250)	488	11.0
Hyperlipidemia (272)	333	7.5
Gout (274)	154	3.5
Ischemic Heart Disease (410-414)	59	1.3
Cardiovascular disease (430-438)	45	1.0
Atherosclerosis (440)	3	0.06
Diseases of the digestive system (520–579)	750	16.9
Systemic lupus erythematosus (710.0)	116	2.6
Rheumatoid arthritis (714, 714.0, 714.2, 714.30-714.33)	15	0.34

**Table 2:** The top ten most commonly used herbal formulas for chronic glomerulonephritis (n = 54726).

Herbal formula	Constituents	Indication of TCM syndrome	Average daily dose (gm/day)	Counts Visit (n)	Prevalence Visits (%)
Jia-Wei-Xiao-Yao-San (JWXYS)	Paeonia lactiflora Pall., Bupleurum chinense DC., Atractylodes macrocephala Koidz., Poria cocos (Schw.) Wolf, Angelica sinensis (Oliv.) Diels., Mentha haplocalyx Briq., Glycyrrhiza uralensis Fisch., Zingiber officinale Rosc., Paeonia suffruticosa Andr., and Gardenia jasminoides Ellis.	Liver and spleen blood deficiency with heat transforming, liver qi stagnation	7.3	32939	60.2
Bu-Yang- Huan-Wu –Tang (BYHWT)	Astragalus membranaceus (Fisch.) Bge., Paeonia veitchii Lynch, Angelica sinensis (Oliv.) Diels., Ligusticum chuanxiong Hort., Carthamus tinctorius L., Prunus persica (L.) Batsch, Pheretima Aspergillum.	Qi deficiency with blood stagnation	4.1	20395	37.3
Du-Huo-Ji-Sheng-ang (DHJST)	Angelica pubescens Maxim, Taxillus chinensis (DC.) Danser, Eucommia ulmoides Oliver, Achyranthes bidentata Blume, Asarum heterotropoides Fr. Schmidt, Gentiana macrophylla Pall, Poria cocos (Schw.) Wolf, Cinnamomum cassia Presl, Saposhnikovia divaricata (Turcz.) Schischek, Ligusticum chuanxiong Hort., Panax ginseng C.A. Mey., Glycyrrhiza uralensis Fisch., Angelica sinensis (Oliv.) Diels., Paeonia lactiflora Pall., Rehmannia glutinosa Libosch.	liver-kidney depletion, dual deficiency of qi and blood, wind-cold-dampness arthralgia	3.2	10393	19.0
Ching-Shin-Lian-Tzyy- Yinn (CSLTY)	Scutellaria baicalensis Georgi, Ophiopogon japonicus, cortex lycii radicis, Plantago asiatica L., Panax ginseng C.A. Mey., Glycyrrhiza uralensis Fisch., Astragalus membranaceus (Fisch.) Bge., Poria cocos (Schw.) Wolf.	hyperactive heart fire, dual deficiency of qi and yin dampness-heat pouring downward	4.1	8264	15.1
Zhi-Bo- Di-Huang- Wan (ZBDHW)	Rehmannia glutinosa Libosch, Dioscorea opposita Thunb., Cornus officinalis Sieb. et Zucc., Poria cocos (Schw.) Wolf, Alisma orientalis (Sam.) Juzep., Paeonia suffruticosa Andr., Anemarrhena asphodeloides, Phellodendron amurense Rupr.	liver-kidney yin deficiency, deficiency fire flaming upward	4.2	7037	12.9
Shu-Jing- Huo-Xue- Tang (SJHXT)	Angelica sinensis, Paeonia lactiflora Pall, Glycyrrhiza uralensis Fisch, Rehmannia glutinosa (Gaertn.) Libosch, Atractylodes Lancea, Achyranthes bidentata Blume, Citrus reticulata Blanco, Prunus persica (L.) Batsch, Clematis chinensis Osbeck, Ligusticum chuanxiong Hort., Stephania tetrandra S. Moore, Notopterygium incisum, Angelica dahurica, Gentiana scabra Bge, Poria cocos, Zingiber officinale Rosc.,	wind-dampness syndrome, blood stasis at meridian and collateral	3.8	6164	11.3
Xiang-Sha- Liu-Jun-Zi- Tang (XSLJZT)	Aquilaria sinensis, Amomum villosum Lour, Citrus reticulata Blanco, Pinellia ternata (Thunb.), Panax ginseng C.A. Mey, Poria cocos, Atractylodes macrocephala Koidz., Glycyrrhiza uralensis Fisch.	spleen-stomach weakness, dampness obstruction with qi stagnation	2.9	5856	10.7
Xiao-Qing-Long-Tang (XQLT)	Ephedra sinica Stapf, Cinnamomum cassia Blume, Paeonia lactiflora Pall, Zingiber officinale Rosc., Asarum heterotropoides Fr. Schmidt, Schisandra chinensis(Turcz.) Baill, Pinellia ternata (Thunb.), Glycyrrhiza uralensis Fisch.	wind-cold fettering the exterior, retained fluid	3.2	4966	9.1
Ma-Zi-Ren-Wan (MZRW)	Cannabis sativa L., Paeonia lactiflora Pall., Citrus aurantium L., Rheum palmatum L., Magnolia officinalis Rehd. et Wils., Prunus armeniaca L. var. ansu Maxim..	Intestinal-stomach dryness-heat, hard bound stool.	1.7	4471	8.2
Ji-Sheng-Shen-Qi-Wan (JSSQW)	Rehmannia glutinosa Libosch, Dioscorea opposita Thunb., Cornus officinalis Sieb. et Zucc., Poria cocos (Schw.) Wolf, Alisma orientalis (Sam.) Juzep., Paeonia suffruticosa Andr., Cinnamomum cassia Blume, Aconitum carmichaelii, Achyranthes bidentata BL., Plantago asiatica L..	kidney yang deficiency, qi transformation abnormal.	4.07	4102	7.5

**Table 3:** The top ten most commonly used single herbs for chronic glomerulonephritis (n = 54726).

**Latin name**	**Indication of TCM syndrome**	Average daily dose (gm)	Counts Visit (n)	Prevalence Visits (%)
Euryale ferox Salisb.	Insecurity of kidney qi, spleen qi deficiency	1.4	5497	10.0
Salvia miltiorrhiza Bunge	Blood stasis, blood heat	1.3	4514	8.2
Coixlacryma-jobi var. ma-yuen (Rom.Caill.) Stapf	Spleen deficiency with dampness encumbrance	1.4	3965	7.2
Astragalus membranaceus (Fisch.) Bge.	Dual deficiency of the lung-spleen, sunken middle qi	1.8	3185	5.8
Achyranthes bidentata Blume	Liver-kidney yin deficiency, deficiency fire flaming upward	1.2	3139	5.7
Sepiella maindroni de Rochebrune	Insecurity of kidney qi, stomach qi ascending counterflow	1.5	2954	5.4
Eucommia ulmoides Oliv	Liver-kidney depletion	1.1	2873	5.2
Imperata cylindrical (L.) Raeusch.	Frenetic movement of blood due to heat, edema.	1.8	2676	4.9
Rehmannia glutinosa Libosch	Liver-kidney yin deficiency, deficiency fire flaming upward	1.7	2516	4.6
Rheum Palmatum L.	Large intestinal heat bind, blood heat, blood stasis	0.6	2016	3.7

**Table 4:** The top ten most frequently used two Chinese herbal products (CHPs) in combination for chronic glomerulonephritis (n = 54726).

**Combination of two CHPs**	**Prescription counts**	**Prevalence (**%**)**
Jia-Wei-Xiao-Yao-San with Bu-Yang- Huan-Wu-Tang	18698	34.2
Jia-Wei-Xiao-Yao-San with Du-Huo-Ji- Sheng-Tang	8684	15.9
Jia-Wei-Xiao-Yao-San with Ching-Shin- Lian-Tzyy- Yinn	7418	13.6
Du-Huo-Ji- Sheng-Tang with Bu-Yang- Huan-Wu -Tang	6425	11.7
Jia-Wei-Xiao-Yao-San with Gorgon	4878	8.9
Jia-Wei-Xiao-Yao-San with Zhi-Bo-Di-Huang- Wan	4499	8.2
Jia-Wei-Xiao-Yao-San with Cordycepssinensis (Berk.) Sacc.	3718	6.8
Bu-Yang- Huan-Wu-Tang with Gorgon	3422	6.3
Bu-Yang-Huan-Wu-Tang with Ching-Shin- Lian-Tzyy-Yinn	3383	6.2
Jia-Wei-Xiao-Yao-San with Coix Seed	3218	5.9

**Table 5:** eGFR (ml/min) analysis results divided into five groups with different stages (n=386).

	eGFR					
Group	number (N)	Before treatment (Mean ±SD)	After treatment within 6 months (Mean ±SD)	P	After treatment within 12 months (Mean ±SD)	P
**Stage 1~2**	71	97.85±65.18	102.5±76.48	0.24	102.19±59.64	0.28
**Stage 3a**	78	51.89±4.39	57.38±10.48	0.00	55.79±12.71	0.01
**Stage 3b**	81	37.67±4.37	41.77±9.87	0.00	40.01±10.87	0.04
**Stage 4**	72	21.8±4.35	24.34±8.03	0.00	21.23±8.79	0.49
**Stage 5**	84	9.12±3.55	12.82±14.78	0.02	11.53±19.09	0.24

*※*P value for the use of CHPs before treatment and after treatment within 3-6-9 months. The P value < 0.05, indicating significant differences.

**Table 6:** The possible mechanisms of Chinese herbal products (CHPs) in treating chronic glomerulonephritis.

CHPs	Active ingredients	Possible mechanisms
Single herb (SH)		
*Salvia miltiorrhiza Bunge (Dan-Shen)*	Salvianolic acid B (SAB) Miltirone Tanshinone IIA	(1) Therapeutically alleviates oxidative stress and inflammatory process via modulating PI3K/Akt signaling pathway on rat model [9]. (2) Inhibits thrombosis formation, platelet aggregation, and activation of PLC/PKC pathway on rat model [10].
*Astragalus membranaceus (Huang-Qi)*	Saponins (astragalosides) Flavonoid Polysaccharides	(1) Protection against heart, brain, kidney, intestine, liver, and lung injury in various models of oxidative stress-related disease [11]. (2) Reducing proteinuria and increasing haemoglobin and serum albumin [12].
*Rheum Palmatum L. (Da-Huang)*	Emodin	(1) Suppresses fibronectin and collagen type III expression via the inhibition of ERK1/2 and p38 phosphorylation in TGF-*β*1-stimulated NRK-49F cells [13]. (2) Inhibits renal fibrosis and inflammation to repress the deterioration of renal function [14, 15].
Herbal formula (HF)		
Jia-Wei-Xiao-Yao-San		(1) Reduces proinflammatory cytokine [16]. (2) Improves the survival rate of type 2 diabetes patients with HTN [17]. (3) IL-6 related antidepressant effect on menopausal women with mood disorder [18].
Bu-Yang-Huan-Wu-Tang		(1) Suppresses the inflammatory cytokines and reactive oxygen species in the kidney of rats after induction of brain death [19]. (2) Lowers the index number of kidney hypertrophy (KW/BW) and the alleviate injury of the rat's kidney induced by proteinuria, lowers the quantity of nitric oxide, and reduces the damage of the kidneys, downregulates the expression of ICAM-1,VCAM-1 in renal tissue on diabetic kidney disease rats model [20]. (3) Inhibits angiogenesis and metastasis by affecting the expression levels of VEGF, RGS-5, and HIF-1*α* on nude mice model [21].
Du-Huo-Ji-Sheng-Tang		(1) Anti-inflammatory effect and strikingly suppressed the expressions of Rho/NF-*κ*B signaling pathways as well as TGF-*β*/smad-3 pathways on rat model [22, 23]. (2) Promotes osteogenic differentiation and decreases the aging process of human mesenchymal stem cells in vitro model [24].
Zhi-Bo-Di-Huang-Wan		(1) Enriches neuroendocrine immune pathways for immune-modulatory effects thus resulting in nephritis improvement [6, 25]. (2) Antioxidant capacity by increasing SOD and GSH [26].
Shu-jing-huo-xue-tang		(1) Antihypersensitivity effects by actions on alpha-2 adrenoreceptors in CCI-neuropathic rats [27]. (2) Increased blood circulation on rats model [28].

## Data Availability

The data used to support the findings of this study are included within the article.

## Abbreviations

CHPs: Chinese herbal products
CKD: Chronic kidney disease
CGN: Chronic glomerulonephritis
TCM: Traditional Chinese medicine
CGMH: Chang Gung Memorial Hospital
ICD-9: International Classification of Disease Ninth Revision
HF: Herbal formula
SH: Single herb
JWXYS: Jia-Wei-Xiao-Yao-San
BYHWT: Bu-Yang-Huan-Wu-Tang
KDOQI: Kidney Disease Outcomes Quality Initiative
ACEi: Angiotensin converting enzyme inhibitors
ARB: Angiotensin II receptor blockers
LWDHW: Liu-Wei-Di-Huang-Wan
GMP: Good Manufacturing Practice
ARM: Association rule mining
GI: Gastrointestinal
NO: Nitric oxide
ESRD: End stage renal disease
AKI: Acute kidney injury.
